# Editorial: Insights in life-course epidemiology and social inequalities: 2021

**DOI:** 10.3389/fpubh.2022.980547

**Published:** 2022-07-29

**Authors:** Cyrille Delpierre, Hilde Langseth

**Affiliations:** ^1^CERPOP, UMR 1295, Inserm, Paul Sabatier University, UPS, Toulouse, France; ^2^Department of Research, Cancer Registry of Norway, Oslo, Norway; ^3^Department of Epidemiology and Biostatistics, Imperial College London, London, United Kingdom

**Keywords:** social determinants, quality data, methodological approaches, life-course epidemiology, social inequalities

The aim of this Research Topic was to focus on important research challenges in the field of life course epidemiology and social inequalities in health. This volume gathers thirteen papers dealing with aspects that represent current and future challenges in this field of research. More specifically (1) determinants beyond social position and in new contexts, (2) access to high quality data and biospecimens and how to share harmonized datasets, (3) methodological approaches to analyse complex datasets from different sources.

Which social and living conditions that may act as a mechanism to explain the construction of social inequalities over the life course is an important challenge. The paper by Dong et al. highlights the importance of focus on childhood conditions, in particular child malnutrition, which is common in developing countries, as well as developed countries. This is particularly in challenging in context of the global warming. Based on more than 13,000 elderly Chinese people aged 65–99 years, the authors show that childhood starvation is associated with socioeconomic determinants (age, gender, residency, education level, income level) and find a persistent negative cumulative effect of childhood starvation on the quantity and quality of life. Haugland et al. reminds us of the importance of Adverse Childhood Experiences (ACEs) as exposures that should be more targeted by public health strategies. This study including 28,047 adults, shows that the prevalence of ACEs (family conflict, lack of adult support, struggle with bad memories, and difficult childhood) varies with socio-demographic factors (age, gender, marital status, and history of divorced parents) and that exposure to ACEs is associated with low socio-economic status in adulthood (low educational attainment, perceived financial difficulties, receipt of social benefits).

The influence of ethnicity on health is a well-known but the drivers behind this remain less understood. The systematic review by Rubin et al. examines the potential role of genetics by analyzing the research that has been published in the US about the genetic factors of Alzheimer's Disease and Related Dementias (ADRDs) among racial/ethnic minorities, which are disproportionality affected. This review of 66 articles highlights that well-established ADRD genetic risk factors for Caucasian populations have not been studied to the same degree in minority U.S. populations which are underrepresented. The study of Athavale et al. analyse the social conditions associated with ethnicity and show that infection and mortality due to COVID-19 infection among Non-Hispanic Black (NHB) and Hispanics was considerably higher than Non-Hispanic White (NHW) mainly because of social unfavorable conditions more likely to concern racial minorities poverty.

Social inequalities in health are not just an issue in high-income countries. The study of Akokuwebe et al. highlights the need to analyze social inequalities in low-income countries as well. The increased burden of non-communicable disease and the double burden of malnutrition (undernutrition and over-nutrition) in low-income countries due to the epidemiological transition have become a public health concern, which has not received the attention that this problem deserves. This study established the synchrony of a double burden of underweight and overweight/obesity among 3,263 women of reproductive age in South Africa, although the prevalence of underweight was declining, while overweight/obesity increased significantly over the study period. It also highlights the influence of social determinants (age, marital status, education, employment status, wealth, ethnicity, and residence), with more advantaged people more likely to be overweight/obese and people from rural areas or of non-African/Black ethnicity more likely to be underweight. Farias et al. describes the temporal trend of stomach cancer mortality in Brazil which is a middle-income country characterized by great internal socioeconomic heterogeneity. The study shows a decline in stomach cancer over time with periods of variation similar to the behavior observed in both high and low-income countries. The findings point to the need of understanding the behavior of stomach cancer mortality in different geographical regions, since they present different socioeconomic characteristics.

Investigating the complex interplay between life-course exposures and disease requires access to high quality data and biospecimens. In the last decades there has been large initiatives in establishing cohort studies across the world, however the efforts to make the collected data available to the scientific community has been sparse. In a data report by Rodriguesz-Laso et al. they provided a map of initiatives that harmonize patient cohorts across the world. Most initiatives are partnered with universities, hospitals and research institutions. The paper focus on the strengths of integration of cohort studies to take the advantage of already collected information to increase the sample size in studies of uncommon exposures, rare diseases, less strong associations, or very restricted populations like in personalized medicine. O'Leary et al. write about development of a multi-study repository to support research on veteran health. The study aimed to describe the selection of studies included in the repository, the design of metadata-driven architecture for secure storing and tracking of data and biospecimens and development of a process to review the scientific and ethical merit of data and specimen request. This is a good example of the importance of using data and biospecimens from several cohorts to get sufficient statistical power to study rare exposures. This multi-study repository provides a structure that can be used to support the sharing of data and specimens across multiple content areas for different types of research studies.

Research in life course epidemiology also raises important methodological issues. In particular, the question of causality is a major challenge in observational studies. Three studies use Mendelian randomization (MR) to analyze causal effects of their exposure on health outcomes. The study of Probst-Hensch et al. examines the effect of BMI on lung function (LF). A negative causal BMI LF effect is observed with a stronger effect for childhood BMI highlighting the importance of a life course perspective in studies using MR method. The two other studies use MR to analyze the causal effect of education on health. The study of Yoshikawa et al. which is one of the first investigating the association between education and COVID-19 severity, shows that education is associated with a lower risk of COVID-19 severity. The study of Wang et al. investigates the causal effect of education on 14 urological and reproductive health outcomes. Education is associated with a higher or lower risk according to health outcomes. However, in both studies, the mechanisms underlying the associations found are unknown and unexplored. Furthermore, the SNPs used as instrumental variables for education are different between the two studies, raising questions that deserve further investigation about how best to use (if relevant) MR for analyzing social traits. Other approaches may be relevant especially when we are interested in mechanisms and multiple mediators may exist, a situation that is not uncommon in life course epidemiology. Tai et al. proposes a method using G-computation algorithm to conduct causal mediation analysis in the presence of multiple ordered mediators. Their approach is powerful and versatile for settings with multiple mediators. An application of the method is proposed to investigate the mediating role of early and late hepatitis B virus (HBV) viral load in the effect of hepatitis C virus (HCV) infection on hepatocellular carcinoma (HCC). Another methodological issue is no longer causality but prediction, in particular how best to predict a disease using the large amount of data available in the most relevant way. Lufkin et al. proposes a Bayesian regression model to characterize the risk of Rheumatoid Arthritis (RA) from common comorbidities, demographic, socioeconomic, and behavioral factors that are known to associate with RA. The model demonstrates a high predictive accuracy in comparison with other models reported in the literature and model is able to identify important second- and third-order interactions between the risk factors, which may have important clinical relevance and stimulate further research to understand the mechanisms underlying such interactions.

In summary we hope that the papers put together in this Research Topic will be helpful and raise awareness on important scientific challenges and opportunities in future life course epidemiology research.

## Author contributions

CD and HL contributed as monitoring editors for this Research Topic. Both authors listed have made a substantial, direct, and intellectual contribution to the work and approved it for publication.

## Conflict of interest

The authors declare that the research was conducted in the absence of any commercial or financial relationships that could be construed as a potential conflict of interest.

## Publisher's note

All claims expressed in this article are solely those of the authors and do not necessarily represent those of their affiliated organizations, or those of the publisher, the editors and the reviewers. Any product that may be evaluated in this article, or claim that may be made by its manufacturer, is not guaranteed or endorsed by the publisher.

